# Keeping Fruit and Vegetables

**Published:** 1893-04

**Authors:** 


### KEEPING FRUIT AND VEGETABLES.

It is surely of as much consequence to know how to keep fruits and vegetables as it is to know how to produce them, and yearly more and more thought and attention are bestowed on the subject of their preservation. It appears that experiments in France have shown that fruits and vegetables stored under ordinary conditions, but heavily dusted with lime, will resist decay for a long time. Potatoes layered in lime kept for fourteen months, and were in as good condition as when dug. Beets, onions, apples, grapes, and quinces similarly treated kept well for varying periods, but all for several months longer than they would have done ordinarily. The lime keeps away moisture, prevents the fruit from absorbing unpleasant odors, and destroys any microbes that may have found a resting place upon the skin or about the stem. This is a preventive within reach of all, and much cheaper than cold storage.—*Mirror and Farmer.*

